# Customer agility, market orientation, and brand image in the context of Chinese market

**DOI:** 10.3389/fpsyg.2022.1062643

**Published:** 2022-12-05

**Authors:** Wang Junfeng, Yang Zesheng, Lai RuQiang

**Affiliations:** ^1^School of Tourism and Hotel Management, Autonomous University of Barcelona, Barcelona, Spain; ^2^Department of Audiovisual Communication and Advertising, Autonomous University of Barcelona, Barcelona, Spain; ^3^Faculty of Business and Economics, University of Malaya, Kuala Lumpur, Malaysia

**Keywords:** customer agility, market orientation, brand image, PLS-SEM, customer-sensing capability, customer-responding capability

## Abstract

**Introduction:**

Customer agility (CA) and market orientation have been widely discussed in prior research. Also, the role of brand image in business making-decision has been emphasized. However, the current analysis lacks integrating the relationship among the three concepts. Thus, this study creatively creates a causal relationship model of CA, market orientation, and brand image.

**Methods:**

Finally, 289 valid samples from the managers in Chinese market was collected for further analysis by partial least squares structural equation modeling (PLS-SEM).

**Results:**

The results show that market orientation can positively affect CA (both customer-sensing capability and customer-responding capability), and CA will further have a positive influence on brand image. Also, market orientation has a direct impact on brand image. Additionally, CA (both customer-sensing capability and customer-responding capability) will mediate the impact of market orientation on brand image.

**Discussion:**

The research has both theoretical and practical contributions. From the theoretical perspective, the results contribute to enriching the brand theory, strategy management, and marketing research. From the practical perspective, the investigation enlightens managers to pay attention to the roles of CA implementation and market orientation strategies in enhancing their brand image.

## Introduction

Customer agility (CA), as a dynamic capability to maintain the competitive advantage of an enterprise, plays an increasingly prominent role in the practical business activities. The current research has focused on some key antecedents of CA, such as technology capability, knowledge management, and organizational structure (Roberts and Grover, 2012a; Chatfield and Reddick, 2018; Panda and Rath, 2021), and its consequences, such as firm performance and competitive activities (Roberts and Grover, 2012b). As the essence of customer-centered concept, CA implementation and its effectiveness have a profound impact on market-perspective and customer-perspective performance (Ngo and Vu, 2020; Wamba, 2022). However, the prior CA research framework has not been systematized, and does not appear in business analysis as a broader concept.

The positive impact of market orientation (MO) on related agility (e.g., organizational agility, supply chain agility, business process agility, strategic agility) has been confirmed by several investigations (e.g., Kanten et al., 2017; Kurniawan et al., 2020a; Alobaidi, 2021; Suprapti and Suparmi, 2022). However, the driving role of market orientation on CA has not been specifically presented. The agility literature has revealed the homogeneity of CA and related agility. Therefore, it implies that the market orientation can become the antecedent of *CA.* Market orientation is considered as one of strategic orientations, which has a strong impact on organizational performance (Butkouskaya and Llonch-Andreu, 2021). Prior studies emphasize the cruciality of market orientation as its definition clearly explains that it is an organizational ability to perceive multi-dimensional market information (customers, competitors, and environmental trends; Kurniawan et al., 2020a; Nurcholis, 2020). Market orientation can significantly improve the organizations’ market performance depending on the sensitive reactions caused to the market changes (Suprapti and Suparmi, 2022).

Also, as a customer-related concept, brand image is rarely associated with agility. The brand literature pays more attention to the relationship between brand image and brand trust, brand loyalty and brand equity. More specifically, a large number of surveys have confirmed the positive impact of brand image on brand equity (Lee et al., 2014). Brand image is also one component of brand equity (Sasmita and Suki, 2015). Brand trust and brand loyalty can be considered as drivers of brand image (Alhaddad, 2015). Brand image refers to consumers’ long-term memory and preference for a brand (Faircloth et al., 2001). It will affect consumers’ purchase decisions (Waluya et al., 2019). From an enterprise perspective, it helps the company host new brands and pick up the sales of current brands (Lee et al., 2011). To some extent, brand image determines firms’ performance (Zhang, 2015). Therefore, this paper investigates brand image as the outcome of CA implementations. Previous studies have investigated the impact of organizational agility on brand image.

Additionally, market orientation is widely applied in brand theory (Iyer et al., 2019). However, most studies focus on the role of market orientation towards enhancing brand performance (O'Cass and Ngo, 2007). As an organizational strategic strategy, market orientation can help consumers deepen their memory of the brand, form a good impression, and ultimately improve the enterprises’ performance through integrated marketing communication channels (Butkouskaya and Llonch-Andreu, 2021). The organization’s market orientation strategies are conducive to guiding consumers to form a good brand image (Urde et al., 2013). Accordingly, this study demonstrates market orientation as another pre-influencing factor to improve brand image.

This study contributes to expand the CA implementation in the brand theory by combining the CA concept with brand image. Also, the investigation contributes to enrich the marketing and communication research through closing the gap of introducing market orientation as the antecedent of brand image. From the managerial perspective, the research stimulates managers to focus more on the role of CA practices in the brand management and decision-making process. Moreover, market orientation strategies should be emphasized together in the CA implementation and brand image maintenance.

## Literature review and hypothesis development

### Customer agility

Agility is described by Ngo and Vu (2020) as a few groups of particular corporate procedures that identify environmental changes and respond quickly and successfully. CA is fundamentally about “deploying resources to respond to rapidly changing market conditions and developing routines in pursuit of improved effectiveness in responses to emerging demands” (Zhou et al., 2018, p. 515). It is a customer-centered dynamic capability of an organization (Roberts and Grover, 2012a). Its ultimate purpose is to quickly grasp customers’ need changes and flexibly meet their demands grasp through CA activities implementation (Ngo and Vu, 2020). Many resources of enterprises will be invested and integrated to address the core needs of consumers (Zhou et al., 2018). It is consistent of customer-sensing capability and customer-responding capability (Roberts and Grover, 2012a). The effectiveness of CA implementations determines firms’ performance and their competitive advantages (Roberts and Grover, 2012b). Essentially, CA has similar attributes and characteristics with other related agility, such as supply chain agility, strategic agility, organizational agility, business process agility, etc.

### Market orientation

Market orientation is conceptualized by Jaworski and Kohli (1993) as a strategic business orientation, including intelligence generation, intelligence dissemination and coordinated action. It presents an organization’s ability to test the changes in market conditions and address these dynamics timely to sustain its performance (Mandal and Saravanan, 2019). Therefore, market-oriented companies should specially focus on the market orientation role in the intricate business environment (Butkouskaya and Llonch-Andreu, 2021).

### Brand image

Brand image is explained as “how a brand is perceived by consumers” (Aaker, 1996, p. 71). It is about “the mental representation of the brand based on individual consumer’s beliefs, ideas and impression” (Malik et al., 2012, p. 13069). The brand image plays a key role in the development of a brand, as it directly influences consumers’ trust in the brand and determines their purchase intentions (Wijaya, 2013). More and more companies are focusing on cultivating and enhancing their brand image due to its strategic role in business operation and its significant impact on firm performance (Malik et al., 2012).

### Customer agility and market orientation

Previous research has confirmed the positive impacts of market orientation on related agility. For instance, Alobaidi (2021) proves that market orientation positively affects supply chain agility. The conclusion from Kurniawan et al. (2020a) demonstrates that organizations can improve their business process agility using the centrality of market orientation by orchestrating internal and external resources. Additionally, Nurcholis (2020) clarifies that market orientation has a direct and positive effect on firms’ agility. Extra evidence from survey of Kurniawan et al. (2020b) uncovers that market orientation will drive organizations’ strategic agility. According to the similarity between CA and related agility (e.g., supply chain agility; business process agility; strategic agility), the following hypothesis could be accordingly stated:

H1: Market orientation positively affects CA.

H1a: Market orientation positively affects customer-sensing capability.

H1b: Market orientation positively affects customer-responding capability.

### Customer agility and brand image

The association between agility and image has been discussed in several surveys. For example, Yi (2008) finds a close connection between firms’ agility and corporate image. Mohammad Shafiee and Ahmadzadeh (2017) confirm the positive impact of organizational agility on brand image. Additionally, as a customer-centric capability, customer agility aims to meet customers’ demands (Roberts and Grover, 2012b). Conversely, once customers’ needs are met, they will create good impression of the brand or products. And according to the similarity between CA and organizational agility, the following hypothesis could be accordingly stated:

H2: CA positively affects brand image.

H2a: Customer-sensing capability positively affects brand image.

H2b: Customer-responding capability positively affects brand image.

Thus, the existence of the above-mentioned relationship between market orientation and brand image may have a further indirect impact on the CA implementation. Moreover, several direct evidence suggest the mediation role of strategic agility in the relationship between market orientation and firm performance (Kurniawan et al., 2020a). The results from Nurcholis (2020) shows that firms’ agility plays a mediating role in the effect of market orientation on marketing performance. According to the similarity between CA and related agility, the following hypothesis could be accordingly stated:

H3: CA mediates the relationship between market orientation and brand image.

H3a: Customer-sensing capability mediates the relationship between market orientation and brand image.

H3b: Customer-responding capability mediates the relationship between market orientation and brand image.

### Brand image and market orientation

Market orientation can be the driver of brand performance (O'Cass and Ngo, 2007). Iyer et al. (2019) point out that market orientation is a brand image-based strategy. Also, market orientation can significantly enhance brand equity (Ind and Bjerke, 2007). While brand image is a key component of brand equity (Urde et al., 2013). Thus, the following hypothesis could be accordingly stated:

H4: Market orientation positively affects brand image.

**Figure 1:**
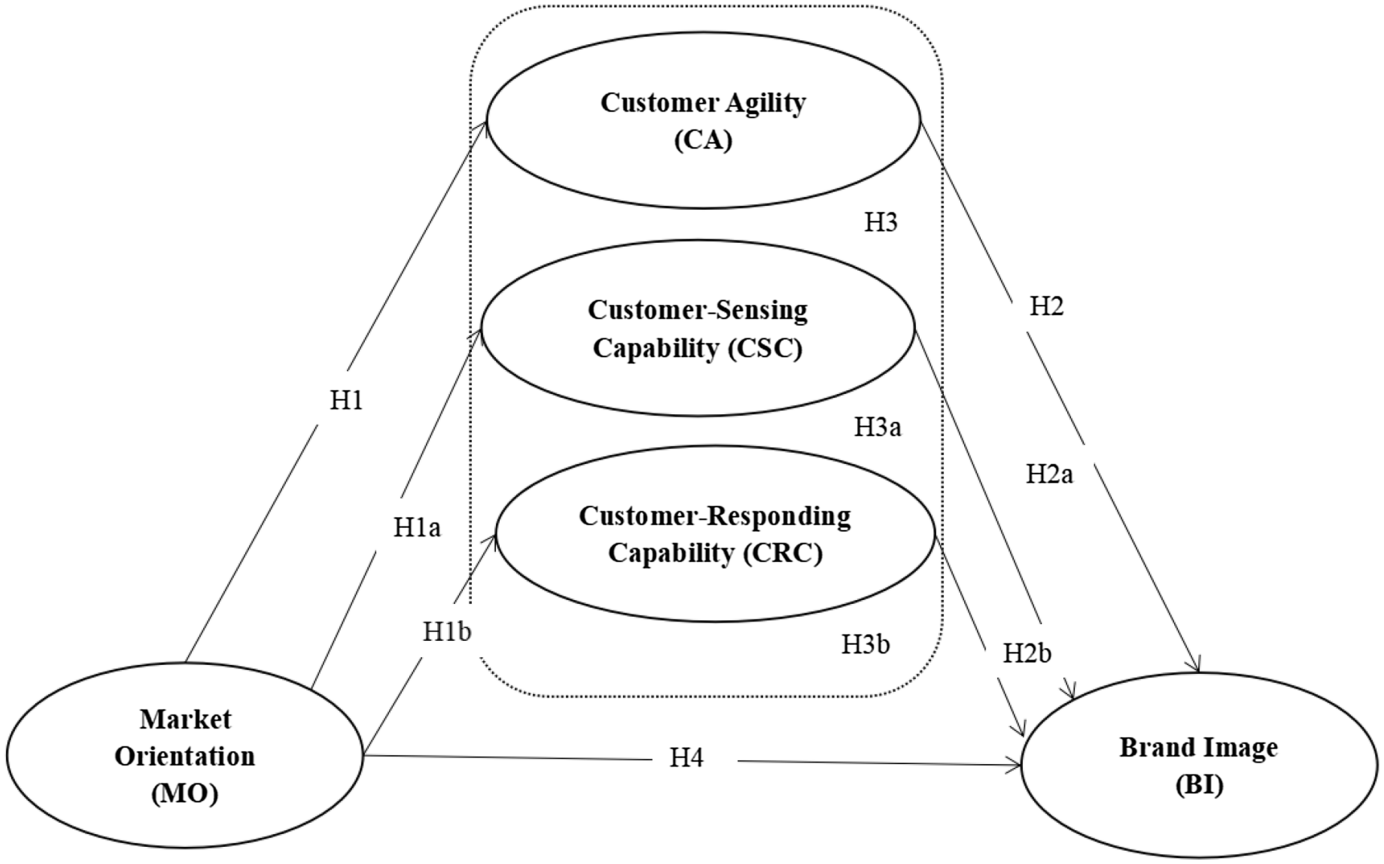
Research model.

Based on the aforementioned hypotheses and the relationships among various constructs, the following research framework is developed (Figure 1).

## Methodology

### Research design, data collection and sample information

The survey is conducted *via* the electronic questionnaire form for delivery (Lewis, 1995). Electronic questionnaire is widely applied in current academic research because of their efficiency, convenience and low cost (Wright, 2005). In order for respondents to fill in items more accurately, some terms (such as CA, market orientation, etc.) are explained in the beginning of the questionnaire. Also, respondents can ask about items that they have difficulty understanding through the message function of the electronic questionnaire. To increase the response rate, each respondent will receive a monetary reward for completing the questionnaire. The respondents are targeted at enterprise managers in Chinese market. The original questionnaire is created in English based on previous literature. Then it is translated in Chinese to make respondents better understand the items. Through back translation, no wording issues are identified. Before official delivery, six experts from business discipline are invited to recheck the questionnaire. The questionnaire is issued from August 1, 2022, to September 1, 2022. A total of 300 questionnaires are delivered and finally, 289 valid questionnaires are collected for further data analysis. Table 1 presents the respondents’ profile information, including age, educational background, marital status, gender, geographical origin, working years and industry. The characteristics of the sample information are generally consistent with the current demographic and social characteristics of China.

**Table 1:** Sample information.

Respondents	Items	Percentage	*N*	Respondents	Items	Percentage	*N*
Gender	Male	46.71%	135	Education	Elementary school	4.50%	13
	Female	53.29%	154		Middle school	15.57%	45
	Total	100%	289		High school	23.53%	68
Age	Under 18 years old	0.35%	1		College	24.22%	70
	18–25 years old	19.38	56		Bachelor’s degree	22.49%	65
	26–30 years old	20.76%	60		Master’s degree	8.65%	25
	31–40 years old	15.57%	45		Doctoral degree	1.04%	3
	41–50 years old	24.91%	72		Total	100%	289
	51–60 years old	11.07%	32	Working years	Less than 1 year	4.15%	12
	60 years old and above	7.96%	23		1–3 years	24.22%	70
	Total	100%	289		3–5 years	19.38%	56
Marital status	Unmarried	41.52%	120		5–10 years	30.80%	89
	Married	58.48%	169		More than 10 years	21.45%	62
	Total	100%	289		Total	100%	289
Geographical origin	East China	19.03%	55	Industry in which you work	Manufacturing industry		104
	West China	21.11%	61		Service industry		180
	South China	16.61%	48		Others		5
	North China	43.25%	125		Total		289
	Total	100%	289				

*N*, number of respondents.

### Measurement

The Likert 5-point scale is applied in the research (Joshi et al., 2015). The scales of Kohli et al. (1993) are adopted for the measures of market orientation. The measurement items of CA come from Roberts and Grover (2012a,b). Brand image mainly draws on the measurement criterion of Hien et al. (2020). The full list of scales used is presented in Table 2.

**Table 2:** Reliability and convergent validity tests of variables.

Variables	Items	Loadings
Customer-sensing capability (CSC) (Roberts and Grover, 2012a,b) *α* = 0.958; C.R. = 0.967; AVE = 0.856		
CSC1	We continuously try to discover additional needs of our customers of which they are unaware.	0.913
CSC2	We extrapolate key trends to gain insight into what users in a current market will need in the future.	0.891
CSC3	We continuously try to anticipate our customers’ needs even before they are aware of them.	0.898
CSC4	We attempt to develop new ways of looking at customers and their needs.	0.960
CSC5	We sense our customers’ needs even before they are aware of them.	0.961
Customer-responding capability (CRC) (Roberts and Grover, 2012a,b) *α* = 0.878; C.R. = 0.911; AVE = 0.671		
CRC1	We respond rapidly if something important happens concerning our customers.	0.839
CRC2	We quickly implement our planned activities concerning customers.	0.818
CRC3	We quickly react to fundamental changes concerning our customers.	0.821
CRC4	When we identify a new customer need, we are quick to respond to it.	0.819
CRC5	We are fast to respond to changes in our customers’ product or service needs.	0.794
Market orientation (MO) (Kohli et al., 1993) *α* = 0.987; C.R. = 0.987; AVE = 0.709		
MO1	In this business unit, we meet with customers at least once a year to find out what products or services they will need in the future.	0.803
MO2	Individuals from our manufacturing department interact directly with customers to learn how to serve them better.	0.839
MO3	In this business unit, we do a lot of in-house market research.	0.870
MO4	We are slow to detect changes in our customers’ product preferences.	0.868
MO5	We poll end users at least once a year to assess the quality of our products and services.	0.812
MO6	We often talk with or survey those who can influence our end users’ purchases (e.g., retailers, distributors).	0.797
MO7	We collect industry information by informal means (e.g., lunch with industry friends, talks with trade partners).	0.835
MO8	In our business unit, intelligence on our competitors is generated independently by several departments.	0.857
MO9	We are slow to detect fundamental shifts in our industry (e.g., competition, technology, regulation).	0.862
MO10	We periodically review the likely effect of changes in our business environment (e.g., regulation) on customers.	0.803
MO11	A lot of informal “hall talk” in this business unit concerns our competitors’ tactics or strategies.	0.873
MO12	We have interdepartmental meetings at least once a quarter to discuss market trends and developments.	0.864
MO13	Marketing personnel in our business unit spend time discussing customers’ future needs with other functional departments.	0.815
MO14	Our business unit periodically circulates documents (e.g., reports, newsletters) that provide information on our customers.	0.790
MO15	When something important happens to a major customer of market, the whole business unit knows about it within a short period.	0.836
MO16	Data on customer satisfaction are disseminated at all levels in this business unit on a regular basis.	0.839
MO17	There is minimal communication between marketing and manufacturing departments concerning market developments.	0.870
MO18	When one department finds out something important about competitors, it is slow to alert other departments.	0.864
MO19	It takes us forever to decide how to respond to our competitor’s price changes.	0.812
MO20	Principles of market segmentation drive new product development efforts in this business unit.	0.864
MO21	For one reason or another we tend to ignore changes in our customer’s product or service needs.	0.807
MO22	We periodically review our product development efforts to ensure that they are in line with what customers want.	0.863
MO23	Our business plans are driven more by technological advances than by market research.	0.869
MO24	Several departments get together periodically to plan a response to changes taking place in our business environment.	0.815
MO25	The product lines we sell depends more on internal politics than real market needs.	0.866
MO26	If a major competitor were to launch an intensive campaign targeted at our customers, we would implement a response immediately.	0.816
MO27	The activities of the different departments in this business unit are well coordinated.	0.865
MO28	Customer complaints fall on deaf ears in this business unit.	0.833
MO29	Even if we came up with a great marketing plan, we probably would not be able to implement it in a timely fashion.	0.837
MO30	We are quick to respond to significant changes in our competitors’ pricing structures.	0.866
MO31	When we find out that customers are unhappy with the quality of our service, we take corrective action immediately.	0.865
MO32	When we find that customers would like us to modify a product of service, the departments involved make concerted efforts to do so.	0.862
Brand image (BI) (Hien et al., 2020) *α* = 0.965; C.R. = 0.971; AVE = 0.850		
BI1	This brand’s quality is high.	0.931
BI2	This brand’s features are better than its competitors’ ones.	0.917
BI3	This brand’s characteristics can be distinguished from competitors.	0.925
BI4	This brand does not disappoint its customers.	0.923
BI5	It is one of the best brands in the industry.	0.917
BI6	This brand is stable in the mark.	0.918

α, Cronbach’s alpha; C.R., composite reliability; AVE, average variance extracted.

### Data analysis

This study will apply partial least squares structural equation modeling (PLS-SEM) to test the hypotheses with SmartPLS 3 (Hair et al., 2019). The Two-step PLS approach will be conducted, consists of estimation of the measurement model and examination of the structural model. The final fitting indexes (SRMR, duls, dg) of original data meet the standardized requirements (Dijkstra and Henseler, 2015). The convergent validity, discriminant validity, and reliability of the scale are calculated for testing the measurement model (Henseler et al., 2016).

## Results

### Model validation

Table 2 shows that Cronbach’s alpha is above 0.7, composite reliability (CR) values are greater than 0.7, average extracted variance (AVE) values are higher than 0.5, and the outer loadings are above 0.7 (Table 2). All the results comply with the standards. Then, the structural model is examined through the bootstrap resampling program.

The discriminant validity of the scale is shown in Table 3. The average extraction variance of each variable is greater than the correlation coefficient between this and other variables, indicating that the scale’s discriminant validity is also acceptable.

**Table 3:** Results of discriminant validity tests of the variables.

	MO	CSC	CRC	BI
Market orientation (MO)	0.842			
Customer-sensing capability (CSC)	0.220	0.925		
Customer-responding capability (CRC)	0.268	0.230	0.819	
Brand image (BI)	0.205	0.223	0.273	0.922

### Estimation of research model

From the values of path coefficients (estimate) and significance levels (C.R.) of each latent variable in the results of structural equation modelling analysis, it is possible to determine the causal relationship and influence strength between various factors and test the overall path hypothesis proposed above (see Table 4). Among the seven hypothesized paths, the standardized path coefficients of five paths (including MO-- > CSC; MO-- > CRC; MO-- > CA; CRC-- > BI; CA-- > BI) passed the significance test of 0.001 level. The standardized path coefficients of these five paths are 0.220, 0.268, 0.310, 0.207, and 0.278, respectively. Specifically, **H1a, H1b, H1, H2a, and H2** passed the significance test of 0.001. Meanwhile, the standardized path coefficients of the two paths (CSC-- > BI; MO-- > BI) passed the significance test of 0.05 level, 0.149, and 0.117, respectively. Their *p*-values are 0.010 and 0.046, respectively. Therefore, the empirical data support **H2a** and **H4**.

**Table 4:** Results of the structural equation modelling test.

Path	Estimate	S.E.	*T* value	*P*	Conclusion
MO-- > CSC	0.220	0.059	3.741	0.000***	H1a Supported
MO-- > CRC	0.268	0.059	4.546	0.000***	H1b Supported
MO-- > CA	0.310	0.059	5.251	0.000***	H1 Supported
CSC-- > BI	0.149	0.058	2.581	0.010*	H2a Supported
CRC-- > BI	0.207	0.059	3.488	0.000***	H2b Supported
CA-- > BI	0.278	0.060	4.602	0.000***	H2 Supported
MO-- > BI	0.117	0.058	1.999	0.046*	H4 Supported

MO, Market orientation; CSC, Customer-sensing capability; CRC, Customer-responding capability; CA, Customer agility; BI, Brand image. *** indicates *p* < 0.001; * indicates *p* < 0.05.

### Mediation test

In this study, the procedure proposed by MacKinnon et al. (2002) was used to test the mediation effect of customer-sensing capability and customer-responding capability using the Bootstrap method of AMOS software. The sample size was chosen to be 2000, and the mediating effect was judged to be significant based on whether the indirect effect included 0 at the 95% confidence interval.

The analysis (Table 5) showed that customer-sensing capability mediates the impact of market orientation on brand image with 95% confidence intervals of (0.006; 0.067), excluding 0. The indirect effect of customer-sensing capability is 0.033, which is significant at the level of 0.05. Customer-responding capability also mediates the impact of market orientation on brand image with 95% confidence intervals of (0.021; 0.100), excluding 0. And the indirect effect of customer-responding capability is 0.056, which is significant at the level of 0.01. The mediating effect of customer-responding capability is higher than that of customer-sensing capability. The overall mediating role of CA in the impact of market orientation on brand image is significant, with 95% confidence intervals of (0.043, 0.145), excluding 0. The overall indirect effect of CA is 0.086.

**Table 5:** Bootstrapping effects and 95% confidence intervals (CI) for the mediation model.

	Effect	*P*-value	95% confidence interval		Conclusion
			Lower bound	Upper bound	
MO-- > CSC-- > BI	0.033	0.037	0.006	0.067	H3a Supported
MO-- > CRC-- > BI	0.056	0.007	0.021	0.100	H3b Supported
MO-- > CA-- > BI	0.086	0.001	0.043	0.145	H3 Supported

MO, Market orientation; CSC, Customer-sensing capability; CRC, Customer-responding capability; CA, Customer agility; BI, Brand image.

## Discussion

### Theoretical contributions

Firstly, the impact of market orientation on CA contributes to the strategic management by closing the gap of lacking analysis of the relationship between strategic orientations and firms’ agility. Specifically, this study finds that market orientation will have a positive influence on both customer-sensing capability and customer-responding capability, which extends the conclusion of Roberts and Grover (2012a). This finding demonstrates that market orientation can enhance the effectiveness of CA implementation in the business activities.

Secondly, the data from the survey uncovers that CA positively affects brand image, which contributes to the brand theory. More specifically, both customer-sensing capability and customer-responding capability will positively affect brand image, which is consistent with the findings of Jaworski and Kohli (1993). The results confirm that dynamic capabilities of an organization (e.g., CA) can improve its brand image.

Thirdly, the direct impact of market orientation on brand image confirms the findings of Mohammad Shafiee and Ahmadzadeh (2017), which proves that organizational agility will positively affect brand image. As a strategic concepts, market orientation will help organizations improve their brand image.

Finally, CA also mediates the relationship between market orientation and brand image, which complies with the results of Iyer et al. (2019). That is, when implementing market orientation to improve brand image, organizations should focus on the role of CA. Both customer-sensing capability and customer-responding capability can help organizations to enhance their brand image when applying market orientation strategies. Also, it is necessary to give play to the synergy of customer sensing capability and customer responding capability, rather than separate them.

### Practical contributions

The direct effects of market orientation and CA on brand image imply that managers can improve the image and attitude of consumers towards their brands through implementation of market orientation and CA activities. And the stronger influence of CA on brand image suggests that managers should invest more resources in improving their customer sensing and responding capability. Surely, the role of market orientation should not be ignored. The result that market orientation has a direct and positive impact on CA indicates that managers also need to pay attention to the effectiveness of conducting market orientation activities, because it will promote the improvement of some dynamic capabilities (such as CA) of enterprises. Additionally, the mediating role of CA in the relationship between market orientation and brand image indicates that managers can enhance the efficiency of market orientation towards improving brand image through the implementation of CA activities.

### Research limitations

As with other research, this article also has some limitations. First, the research just estimates the impact of market orientation. Future study can analyze the effects of customer orientation and technology orientation on CA and brand image (Brady and Cronin 2001; Butkouskaya et al., 2021). Second, some moderators (e.g., company size and gender) are ignored in this study. Further analysis can be conducted by introducing these moderators on the basis of our model. Lastly, the investigation is only conducted in one country. In the future, inter-country analysis can be further examined.

## Conclusion

The investigation tests the causal relationship model of market orientation, CA, and brand image. More specifically, this study confirms the positive impact of market orientation on CA, and further the CA implementation role in enhancing brand image. Also, market orientation has a direct effect on brand image. The results show that market orientation positively affect both customer-sensing capability and customer-responding capability. Also, both customer-sensing capability and customer-responding capability can be drivers of brand image. Additionally, both customer-sensing capability and customer-responding capability will mediate the relationship between market orientation and brand image. Totally, CA can be the mediator in the relationship between market orientation and brand image.

This study has both theoretical contributions and managerial contributions. From the theoretical perspective, the study creatively connects the concepts of strategic orientation and agility, which contributes to enriching the strategic management research. The impact of CA and market orientation on brand image contributes to broaden the brand theory. Also, the mediating role of CA in the relationship between market orientation and brand image enlightens future research of the agility topic.

From the managerial perspective, the results enlighten managers should focus on the role of market orientation and CA in the process of optimizing their brand image. More specifically, managers should pay more attention to the cultivation of their customer sensing and responding capabilities. Also, the synergy influence of customer sensing and responding capabilities should be emphasized in the improvement of brand image through the implementation of market orientation activities.

## Data availability statement

The raw data supporting the conclusions of this article will be made available by the authors, without undue reservation.

## Author contributions

WJ: contributes to data collection, data analysis and writing the original manuscript. YZ: helps review and check the final revison. LR: contributes to edit the format of the manuscript. All authors contributed to the article and approved the submitted version.

## Conflict of interest

The authors declare that the research was conducted in the absence of any commercial or financial relationships that could be construed as a potential conflict of interest.

## Publisher’s note

All claims expressed in this article are solely those of the authors and do not necessarily represent those of their affiliated organizations, or those of the publisher, the editors and the reviewers. Any product that may be evaluated in this article, or claim that may be made by its manufacturer, is not guaranteed or endorsed by the publisher.
